# Investigating presentations and outcomes of a joint HIV–renal clinic

**DOI:** 10.7448/IAS.17.4.19566

**Published:** 2014-11-02

**Authors:** Jake Scott, Deborah Williams

**Affiliations:** 1 Lawson Unit, Brighton and Sussex Medical School, Brighton, UK; 2 Lawson Unit, Brighton and Sussex University Hospitals, Brighton, UK

## Abstract

**Introduction:**

HIV patients are at risk of renal dysfunction directly from HIV, indirectly from chronic inflammation as well as from antiretroviral drug toxicity. In particular, tenofovir (TDF) has been associated with proximal renal tubular dysfunction. A joint HIV–renal clinic was set up in 2009 to facilitate a timely review and minimize additional follow-up appointments. Brighton has a cohort of 2,150 HIV patients, 90% are on ARVs with 910/1,935 (47.0%) on regimens including TDF. This study aims to investigate the utility of this clinic and describe renal disease in this cohort.

**Materials and Methods:**

Notes of patients scheduled for assessment at the clinic between 2012 and 2014 were reviewed. Demographics, HIV history, number of visits, reason for referral and outcome information were collected.

**Results:**

Sixty-five patients, median age 51 years (28–88) and median duration of HIV 163 months (20–335), were reviewed. Forty-two were taking TDF for a mean of 55.8 months (9–122). Forty-two patients were reviewed once with a median number of visits of 1 (1–4).

Of those on TDF with proteinuria, 5 (13.2%) had TDF toxicity diagnosed and were discontinued at once, 27 continued with close monitoring with a further 7 subsequently discontinuing; total discontinued were 12 (32%). Overall, blood pressure control was the commonest intervention, 23/65 (35.4%), with 8/12 (66.7%) in the non-TDF proteinuria group. Four (6%) patients underwent renal biopsy (2 focal segmental glomerulosclerosis, 1 IgA nephropathy and 1 glomerulonephritis and granuloma). In 4 (6%) creatinine rise was attributed to NSAIDs, creatine or protein supplements usage.

**Conclusions:**

TDF toxicity was the commonest reason for referral but only a minority (13.2%) needed to discontinue immediately. Optimizing BP control was the most frequent outcome suggesting this is an underappreciated cause for renal dysfunction amongst HIV physicians, as were other causes such as creatine and protein supplement usage. Running a “one-stop-shop” clinic supports continuation of tenofovir in patients with proteinuria, and supports the diagnosis and management of poorly controlled hypertension and streamlines the management of these patients.

**Table 1:** Reasons for referral to HIV-renal clinic

Reason for referral	Frequency
Proteinuria on TDF	38 (58%)
Proteinuria not on TDF	12 (18%)
Raised creatinine without proteinuria	15 (23%)

